# Osteopathic Manipulative Treatment to Optimize the Glymphatic Environment in Severe Traumatic Brain Injury Measured With Optic Nerve Sheath Diameter, Intracranial Pressure Monitoring, and Neurological Pupil Index

**DOI:** 10.7759/cureus.13823

**Published:** 2021-03-11

**Authors:** Samir Kashyap, James Brazdzionis, Paras Savla, James A Berry, Saman Farr, Tye Patchana, Gohar Majeed, Hammad Ghanchi, Ira Bowen, Margaret Rose Wacker, Dan E Miulli

**Affiliations:** 1 Neurosurgery, Riverside University Health System Medical Center, Moreno Valley, USA; 2 Neurosurgery, Arrowhead Regional Medical Center, Colton, USA

**Keywords:** omt, glymphatic, onsd, tbi, osteopathic, optic nerve sheath

## Abstract

Background

Traumatic brain injury (TBI) has a complex pathophysiology that has historically been poorly understood. New evidence on the pathophysiology, molecular biology, and diagnostic studies involved in TBI have shed new light on optimizing rehabilitation and recovery. The goal of this study was to assess the effect of osteopathic manipulative treatment (OMT) on peripheral and central glial lymphatics in patients with severe TBI, brain edema, and elevated intracranial pressure (ICP) by measuring changes in several parameters regularly used in management.

Methodology

This was a retrospective study at a level II trauma center that occurred in 2018. The study enrolled patients with TBI, increased ICP, or brain edema who had an external ventricular drain placed. Patients previously underwent a 51-minute treatment with OMT with an established protocol. Patients received 51 minutes of OMT to the head, neck, and peripheral lymphatics. The ICP, cerebrospinal fluid (CSF) drainage, optic nerve sheath diameter (ONSD) measured by ultrasonography, and Neurological Pupil Index (NPi) measured by pupillometer were recorded before, during, and after receiving OMT.

Results

A total of 11 patients were included in the study, and 21 points of data were collected from the patients meeting inclusion criteria who received OMT. There was a mean decrease in the ONSD of 0.62 mm from 6.24 mm to 5.62 mm (P = 0.0001). The mean increase in NPi was 0.18 (P = 0.01). The mean decrease in ICP was 3.33 mmHg (P= 0.0001). There was a significant decrease in CSF output after treatment (P = 0.0001). Each measurement of ICP, ONSD, and NPi demonstrated a decrease in overall CSF volume and pressure after OMT compared to CSF output and ICP prior to OMT.

Conclusions

This study demonstrates that OMT may help optimize glial lymphatic clearance of CSF and improve brain edema, interstitial waste product removal, NPi, ICP, CSF volume, and ONSD. A holistic approach including OMT may be considered to enhance management in TBI patients. As TBI is a spectrum of disease, utilizing similar techniques may be considered for all forms of TBI including concussions and other diseases with brain edema. The results of this study can better inform future trials to specifically study the effectiveness of OMT in post-concussive treatment and in those with mild-to-moderate TBI.

## Introduction

Traumatic brain injury (TBI) is a broad spectrum of disease. In its mildest form, TBI causes a temporary alteration of cognitive function, which can manifest as confusion, amnesia, loss of consciousness, headache, impaired focus, balance, and coordination due to changes in neuronal synaptic transmission, neuronal environmental chemical changes, changes in cerebral blood flow, and brain edema [1]. In its most severe form, patients can be rendered comatose, have increased intracranial pressure (ICP), and even suffer devastating neurological sequelae [1].

Invasive neuromonitoring such as an intraparenchymal probe or external ventricular drain (EVD) have long been the standard method of directly assessing ICP. Ocular ultrasonography assessing the optic nerve sheath diameter (ONSD) and pupillometry with assessment of the Neurological Pupil index (NPi) (NeuroOptics) are noninvasive measures that reliably predict increased ICP [2,3]. The literature reports ranges of normal and abnormal ONSD parameters with a normal diameter less than 6.0 mm which correlates to a normal ICP [4]. The size of the ONSD is physiologically created through the flow of cerebrospinal fluid (CSF) in the narrow trabeculated subarachnoid space that surrounds the anterior segment of optic nerve. This space surrounding the optic nerve expands with increasing ICP. Prolonged distention results in papilledema after hours or days, especially in the case of space-occupying lesions; however, trauma can lead to papilledema in both an acute and chronic fashion with prolonged ICP elevations [5]. However, the ONSD measures changes in real time with any change in ICP. The NPi is rated on a scale of 0 to 5 (abnormal less than 3 and normal 3-5) [3]. The NPi measures the pupil size and reactivity due to function of the optic and ophthalmic cranial nerves. These nerves respond to stimuli such as herniation and third nerve compression at the anatomical level and physiologically to brainstem blood flow and oxygenation which is affected by edema and other mass lesions. Adjunctive therapeutic interventions to optimize glial lymphatic, or glymphatics, edema, metabolic changes, and CSF volume may aid in recovery. These interventions may include osteopathic manipulative treatment (OMT), sleep hygiene, participation in cognitive exercises, and proper nutrition. Each is aimed at clearing cerebral metabolic wastes through treatments designed to promote glymphatic function [6]. Further complicating evaluation of recovery, short-term progress may be limited as the temporal duration of healing after TBI is proportional to the severity of the mechanism of the injury that can be directly graded based on the degree of clinical cognitive impairment [7].

The clinical manifestations of TBI are caused by damage that occurs at a microcellular level, disrupting intricate biochemical and electromagnetic physiological functions [8,9]. At the initial moment of blunt force head impact, the primary insult to the central nervous system (CNS) occurs; however, the secondary components of TBI will further affect the final neurological outcome [10]. Successive trauma, particularly during the acute and subacute phase, can result in prolonged recovery and cause permanent damage to the glymphatics, which may manifest later in life through development of chronic traumatic encephalopathy (CTE), as well as increased Tau proteins and beta amyloid plaques, as seen in tauopathies [11]. Further, cerebral homeostatic function is altered and the brain is most vulnerable to secondary injury during the acute to subacute phase where the repair of cytoarchitectural damage is occurring and there may be evidence of interstitial edema [12]. This damage results in changes in protein content which have begun to be assessed as sensitive biomarkers for TBI-glial fibrillary acid protein (GFAP) and ubiquitin C-terminal hydrolase-L1 (UCH-L1) [13].

Therefore, optimizing the pathophysiological state over the long term may reduce risks of secondary injury and promote healing. These conditions with appropriate control of ICP and maintenance of nutritional status should be maintained throughout recovery to promote optimal recovery. Thus, investigating a holistic approach which may include therapies such as OMT may be beneficial in ensuring appropriate homeostasis for recovery. In this study, we report the use of both invasive and noninvasive measures to assess the effect of OMT on peripheral and central glial lymphatics in patients with severe TBI, elevated ICP, and brain edema. OMT is a useful treatment in that it can be completed with limited resources with very few absolute contraindications. The availability of its use makes it an effective tool to improve patient care without increasing cost or putting the patient in any additional undue stress.

## Materials and methods

This was retrospective review conducted at a level II trauma center in 2018. The study was approved by our center’s institutional review board. All patients included in the study received treatment using our center’s standard protocols of care which may include OMT. Patients met inclusion criteria if they were older than 18 years, had an EVD placed per the Brain Trauma Foundation guidelines, which may include deteriorating neurological condition, cerebral edema, and a Glasgow Coma Scale (GCS) less than or equal to eight. Further, patients met inclusion criteria if they were treated in 2018 with a stereotyped 51-minute OMT treatment, as described below. At our center OMT is commonly performed on our patients on the neurosurgical service, and during the selected 2018 time interval, patients were reliably treated and had a stereotyped protocol utilized leading to its choice in investigation. Exclusion criteria included severe orbito-facial trauma, any mass lesion requiring evacuation, recent decompressive craniectomy, or a history of ventriculoperitoneal shunt. All patients meeting the inclusion criteria had an EVD placed based on clinical presentation and need. EVDs remained open during OMT to allow continued CSF drainage, except for brief periodic closures during treatment to measure accurate ICP values.

Each patient included in the study was allowed up to three treatment cycles on different days, had different drainage heights for their EVD which are placed and weaned based on individual pressures and characteristics of the individual patient according to standard neurosurgical protocol at our center, and may have had differing GCS based on clinical status. Patients were not excluded based on individual GCS scores.

Treatment protocol

Patients were placed supine with their head in neutral position and the head of bed elevated to 30 degrees. The EVD was kept at the level of the external auditory meatus during the entire treatment which is the accepted standard, while an EVD remains in place for drainage and monitoring. The EVD level, ICP values, and CSF output were recorded one hour before starting treatment; at zero, 30, and 60 minutes after the initiation of treatment; and for one hour starting 30 minutes after completion of treatment. The CSF output was recorded to ensure that OMT did not increase drainage and independently mitigate the other results. The patients’ GCS, mean arterial pressure (MAP), respiratory rate (RR), systolic blood pressure (SBP), and NPi were also recorded during the same intervals.

A linear ultrasonography probe was used to conduct all the measurements of the ONSD. The probe was placed on the eye lid to obtain an axial cross-section, and we took care to avoid applying excessive pressure on the globe. The ONSD was measured 3 mm behind the optic disc based on standards set in previous studies [4]. The ONSD was measured prior to treatment and immediately post treatment.

Patients underwent 51 minutes of OMT divided as follows: nine minutes for each treatment of thoracic inlet opening, rib raising, and diaphragmatic release, followed by six minutes of thoracic or pedal pump and nine minutes for the compression of the fourth ventricle (CV4) and facial effleurage. CV4 is a treatment to control the tide of CSF flow by compressing the CV4, leading to a rhythmic balanced interchange between all the fluids of the body. These treatments were selected to optimize lymphatic flow and drainage, which may promote drainage of CSF through interactions with the glymphatic system in the brain, and thus decrease intracranial pressure. Results were recorded for all patients undergoing treatment with each 51-minute treatment represented as a singular data point. Patients may have been included multiple times within the study if they underwent multiple treatments.

## Results

We reviewed a total of 21 data points in 11 patients with ICP monitoring before, during, and after treatment. There was a decrease in the ONSD after all treatments. The mean ONSD pretreatment was 6.24 mm (±1.28 mm) and the mean ONSD posttreatment was 5.62 mm (±1.22 mm), resulting in a mean decrease of 0.62 mm (P = 0.0001). Pretreatment NPi was 3.77, and posttreatment NPi was 3.95. The mean increase in NPi was 0.18 (P = 0.01). The mean pretreatment ICP was 13.48 mmHg (4.53 mmHg) and the mean posttreatment ICP was 10.14 mmHg (3.45 mmHg), yielding a mean decrease in ICP 3.33 mmHg (P = 0.0001). There were no incidents of rebound increases in ICP after any treatment sessions. There was a mean decrease in CSF output of 3.5 mL one hour posttreatment in comparison to one hour before treatment (P = 0.0001). During OMT, there was a nonsignificant mean increase of 2.5 mL CSF output over the course of one hour compared to pretreatment output (P = 0.11). This was not seen in all patients with a decrease in CSF output in eight patients. These results are tabulated in Table 1 and Table 2.

**Table 1:** ICP, ONSD, and NPi After 60 minutes of OMT lymphatic technique. ICP: intracranial pressure; NPi: Neurological Pupil Index; ONSD: optic nerve sheath diameter; osteopathic manipulative treatment

	Pretreatment, mean (SD)	Posttreatment, mean (SD)	Average change	P-Value
ICP (mmHg)	13.48 (4.53); range 9-27	10.14 (3.45); range 7-19	(-) 3.33	0.0001
NPi	3.77; range 0-4.7	3.95; range 0-4.9	0.18	0.01
ONSD (mm)	6.24 (1.28); range 4.45-8.7	5.62 (1.22); range 3.9-7.9	(-) 0.62	0.0001

**Table 2:** Mean CSF output prior to, during, and after treatment with osteopathic manipulative treatment. CSF: cerebrospinal fluid; OMT, osteopathic manipulative treatment

	CSF output (mL)	P-Value
Mean hourly CSF output one hour prior to treatment	10	-
Mean hourly CSF output one hour post treatment	6	-
Difference between average hourly CSF output one hour prior to and one-hour after treatment	4	0.0001
Mean total CSF output within 0-30 minutes of treatment	4.5	-
Mean total CSF output within 30-60 minutes of treatment	5.5	-
Average hourly total CSF output during treatment	12.5	-
Mean change in in hourly CSF output one hour prior to treatment versus during treatment	2.5	0.11

## Discussion

In the acute phase, patients with severe TBI may be monitored with devices such as an EVD or intraparenchymal probe to monitor and control ICP. The NPi and ONSD have been validated in the literature as a reliable noninvasive measures of ICP and were used in this study as additional measurements of ICP [3,4]. The increased need for monitoring and differing monitoring modalities in this population lead to a unique opportunity to obtain qualitative measurements in patients with TBI that can transform the acute care of TBI patients. Changes in ONSD can be observed instantaneously with any changes in ICP [4]. This phenomenon was described by Maissan et al. in their observational study of 18 patients, in whom they showed that ICP correlated with simultaneous changes in the ONSD undergoing invasive ICP monitoring after suffering TBI [2]. Thus, any changes in ICPs will be transmitted simultaneously to the optic nerve sheath if there is no obstruction to CSF flow between the orbital and cranial compartments. In our population, initial ONSDs were abnormally elevated at greater than 6 mm due to increased ICP that was being managed with CSF drainage.

We demonstrated a mean decrease in ONSD of 0.62 mm (P = 0.0001). This correlated with a mean decrease in ICP of 3.33 mmHg (P = 0.0001; r^2^ = 0.31). The consistency between these two results gives further support for the mobilization of lymphatics being useful in decreasing ICP. Furthermore, our ONSD on average decreased to less than 6 mm after treatment which correlates to a normal ONSD.

Pupillometry is another noninvasive method that that has been validated in the literature to strongly correlate with ICP and functional outcomes in TBI [3,13]. Pupillary abnormalities are often one of the first signs of impending ICP crisis and are a sign of impaired brainstem perfusion or cranial nerve compression. The pupillomotor fibers are more sensitive to increased ICP because of their location on the periphery of the oculomotor nerve. If these changes are left untreated or not recognized, they can progress to anisocoria or a nonreactive and dilated pupil, which is a neurosurgical emergency. The NPi is an index of six different parameters measured in pupillary response to light that is scaled into three tiers: normal (3.0-4.9), abnormal (<3), and nonreactive (0). In a prospective analysis of 134 patients, Chen et al. found that the NPi was able to detect pupillary changes almost 16 hours before an ICP peak, making it an early predictor of ICP crisis [3]. Our patients demonstrated increases in their NPi which correlated to improvements in ONSD and decreases in ICP, which suggests that OMT may help reduce ICP through all measures. We hypothesize this may be through improvements in glymphatic function and CSF clearance.

Osteopathic manipulative treatment to improve glymphatic function

The link between the central glial lymphatic system and the peripheral lymphatic system is found within the meninges where CSF is absorbed, and the waste products of the brain parenchyma and CSF move into the fluid channels of the meninges and into the peripheral lymphatic system [14]. Before the 21st century it was believed by most that the CNS was devoid of any lymphatic system as there was no histological evidence of tissue that resembled the peripheral lymphatic system. There was direct evidence of intraparenchymal waste products in the CSF that were being cleared through fluid channels in the meninges; however, the mechanism remained unclear [15]. Until recently, the brain glial lymphatic system, or the “glymphatics,” was thought to account for a minor portion of this CSF drainage. This newly discovered glymphatic system of the CNS demonstrated that astrocytes facilitate the clearance of cerebral parenchymal waste and resultant interstitial brain edema through the pulsations of CSF along perivascular spaces between deep penetrating cerebral blood vessels, leptomeningeal sheaths, and aquaporin-4 (AQP4) water channels that drain into the cervical lymphatics [6,16,17]. Studies performed in AQP4-deficient mice showed increased brain water content and larger increases in ICP after induced vasogenic edema. Plog et al. and Smith et al. further investigated this hypothesis using animal studies, for example, one study used four different methods (AQP4 deletion, cisternotomy, acetazolamide treatment, and sleep deprivation) that resulted in decreased glymphatic clearance of a fluorescent tracer into deep cervical lymphatics with further decreases in levels of serum TBI biomarkers [17,18]. Smith et al. have proposed that the role of AQP4 is not central to the glymphatic hypothesis, and that the tracer studies are dependent on the size of the tracers [18].

The safety profile of OMT in patients with TBI has previously been investigated. In a cohort of 24 patients with severe TBI, Cramer et al. showed that OMT using thoracic and pedal pump lymphatic techniques led to a mean decrease in ICP and concurrent increase in mean cerebral perfusion pressure (CPP) values, which resulted in increased CBF [19]. In a follow-up study by Dreyer et al., the same techniques found a favorable trend in ICP, CPP, and patient outcomes in the OMT cohort; however, they did not reach statistical significance [20]. Neither study mentioned the duration of OMT. Patel and Sabini demonstrated that cranial OMT administered for four weeks was a safe and effective adjunct that reduces post concussive symptoms and accelerates recovery [21]. OMT-induced reduction in ICPs is thought to occur via increased CSF outflow from the cranial cavity in addition to improving glymphatic flow and decreasing interstitial brain edema [21].

Extra precautions in regards to performing OMT were taken to avoid manipulation of the calvarium or cervical spine if there was any suspicion of an unstable fracture or ligamentous injury. Any compression of the jugular veins in the neck was also avoided as jugular venous compression will raise ICP [22].

Our study demonstrates that CSF clearance and presumptive cerebral interstitial waste product removal and the accompanying brain edema decrease is enhanced through 51 minutes of cranial-directed OMT, as evidenced by the statistically significant decreases in ICP and ONSD, along with an increase in NPi.

Osteopathic manipulative treatment to improve intracranial pressure

In patients with severe TBI, ICP, and CPP-directed therapy is based on quantitative information obtained through an invasive ICP pressure monitor such as an EVD. In recent times, the efficacy of various noninvasive methods in predicting increased ICP has been studied, including ONSD and NPi measurements [3,4]. We devised a novel protocol to monitor real-time effects of OMT in promoting increased “glymphatic” drainage from the cranial vault to decrease ICPs. Our patients demonstrated a mean increase in NPi of 0.18 (P = 0.01), which further affirmed a direct correlation between the ICP, ONSD, and NPi measurement. This improvement in ICP is directly measured through the implanted EVD, decrease in ONSD, improvement in pupil NPi, and decreased CSF volume, as evidenced by decreased drainage in CSF through the EVD following treatment. This decreased CSF drainage through the EVD is due to improved internal clearance intracranially. Our data continue to support other studies that ICPs may be beneficially impacted through OMT [19]. We hypothesize our lymphatic-based treatments may have directly improved clearance through the aforementioned glymphatic system. Additional treatments such as CV4 may improve secondary factors involved in treatment of ICP and involved in neuronal recovery. In a review of the efficacy of the CV4 technique across various pathologies, trends were found in the improvement of sleeping patterns on electroencephalogram, autonomic function, and pain reduction [23]. These same types of OMT accordingly are safe and can be used in any patients with brain edema or TBI, whether ranging from mild to severe to similarly enhance glymphatic clearance.

Osteopathic manipulative treatment in post concussive treatment

As evidenced by our data in patients with severe TBI, OMT can be used to mobilize the glymphatic system and decrease brain edema. Concussion, which by definition meets the criteria as a minor TBI, can similarly be treated using these OMT techniques. As concussions have been linked to increased brain edema, improving glymphatic function may similarly decrease edema, and promote clearance of toxic metabolites actively being investigated as markers for TBI [9,21]. Being relatively quick and effective, OMT can supplement the treatment of concussions to great effect. Further studies will be needed to quantify the improvement in symptoms and outcomes in concussions. However, consideration of implementation of OMT for patients with TBI ranging from mild to severe may be considered based on improvements demonstrated in measures of glymphatic and CSF clearance taken in our patient population within the highest severity category to theoretically promote homeostatic conditions for recovery.

Limitations

One limitation of this study was that we only examined the acute care of our patients at a single institution. Currently, there is no long-term follow-up available for these patients to determine whether these interventions have an effect on outcomes other than changes within measures of ICP, clearance of CSF, and glymphatic function. Further long-term side effects were not investigated. Although osteopathic neurosurgical residents are adequately trained on standard OMT techniques and how to measure ONSD, there is no way to eliminate minor variations among providers. Furthermore, there is an element of selection bias within our study as all patients had previously been treated and selected for review based on being treated with a previous protocol. Further studies may also consider evaluation of intrinsic biomarkers before and after treatment to identify if any changes in clearance of these toxic metabolites may be measured.

## Conclusions

OMT in concert with standardize therapies of TBI may optimize cerebral blood flow and improve ICP theorized to be from improvement in glymphatic clearance. This improved clearance may promote a more “normal” condition, promote internal glymphatic clearance of toxic metabolites, and may improve recovery potential. We demonstrated a benefit from OMT lymphatic techniques with improvements in ICP, reduction in ONSD from abnormal to normal, and improved NPi in patients with TBI. These benefits may be considered in extrapolation for the treatment of minor TBI, namely, concussions as well as patients with cerebral edema. Our findings help provide the foundation for larger, randomized trials that may combine OMT with a new multimodal monitoring in patients with severe TBI to optimize the brain environment and prevent secondary injury in these patients.
